# P2RY12-Inhibitors Reduce Cancer-Associated Thrombosis and Tumor Growth in Pancreatic Cancers

**DOI:** 10.3389/fonc.2021.704945

**Published:** 2021-09-13

**Authors:** Ana Luisa Palacios-Acedo, Soraya Mezouar, Diane Mège, Lydie Crescence, Christophe Dubois, Laurence Panicot-Dubois

**Affiliations:** ^1^Aix Marseille Univ, INSERM 1263, INRA 1260, Center for Cardiovascular and Nutrition Research (C2VN), Marseille, France; ^2^Department of Digestive Surgery, Timone University Hospital, Marseille, France

**Keywords:** pancreatic tumors, thrombosis, clopidogrel, aspirin, P2Y12 receptor

## Abstract

Platelet function can be modified by cancer cells to support tumor growth, causing alterations in the delicate hemostatic equilibrium. Cancer-cell and platelet interactions are one of the main pillars of Trousseau’s syndrome: a paraneoplastic syndrome with recurring and migrating episodes of thrombophlebitis. Altogether, this leads to a four-fold risk of thrombotic events in cancer patients, which in turn, portend a poor prognosis. We previously demonstrated that anti-P2RY12 drugs inhibit cancer-associated-thrombosis and formation of tumor metastasis in pancreatic cancer models. Here, we aimed to (1) compare the effects of aspirin and clopidogrel on pancreatic cancer prevention, (2) characterize the effects of clopidogrel (platelet P2RY12 inhibitor) on cancer-associated thrombosis and cancer growth *in vivo*, (3) determine the effect of P2RY12 across different digestive-tract cancers *in vitro*, and (4) analyze the expression pattern of P2RY12 in two different cancer types affecting the digestive system. Clopidogrel treatment resulted in better survival rates with smaller primary tumors and less metastasis than aspirin treatment. Clopidogrel was also more effective than aspirin at dissolving spontaneous endogenous thrombi in our orthotopic advanced cancer mouse model. P2RY12 expression gives pancreatic adenocarcinomas proliferative advantages. In conclusion, we propose the hypothesis that clopidogrel should be further studied to target and prevent Trousseau’s syndrome; as well as diminish cancer growth and spread. However, more studies are required to determine the implicated pathways and effects of these drugs on cancer development.

## Introduction

Platelets are recognized as key players in cancer progression, having a myriad of pro- and anti-tumoral effects (1). They participate in each critical step of cancer progression including tumor growth, angiogenesis; and as we have RECENTLY shown, metastasis (1–4). The interaction between tumor cells and platelets leads to platelet-activation and to a cancer-associated hypercoagulable state (Trousseau’s syndrome). This increases the overall risk of developing thrombotic events, which are the second most common cause of death in oncological patients (after cancer progression) (5). Indeed, studies show that between 20% to 30% of all first venous thrombotic events are cancer-related (6). The onset of Trousseau’s syndrome is associated with an overall worse prognosis and survival of patients (7). Platelets are important mediators in this syndrome, including the formation of deep vein thrombosis and pulmonary embolisms (8).

Pancreatic ductal adenocarcinomas (PDACs) are known for having an important pro-thrombotic clinical component (9). This cancer type represents nearly 85% of all pancreatic cancer diagnoses and is very aggressive, with a 5-year net survival rate of 10% (10, 11). In a worrisome manner, the incidence of this type of cancer has been steadily growing, particularly in developed countries (11, 12).

Among the available anti-platelet drugs, P2RY12 antagonists and Aspirin appear to be of particular interest for the treatment of Trousseau’s syndrome, due to their safety and relevant mechanism of action (13). Aspirin prevents the formation of thromboxane A2 (TXA2), a platelet agonist, and at a high concentration, may also limit inflammation (14–16). Different retrospective studies have shown the benefit of long-term Aspirin treatment on digestive-tract cancer development and formation of distant metastasis (17, 18).

Anti-P2RY12 drugs, like clopidogrel, inhibit platelet P2RY12-binding of ADP and, thus, platelet activation (13). ADP plays a crucial role in platelet activation and aggregation, granule secretion, activation of integrin αIIbβ3, and thrombosis (1, 19). During cancer progression, ADP can be released from activated platelet granules, endothelial cells, and cancer cells themselves (20, 21). The released ADP will also enhance the tumor-cell induced platelet aggregation (TCIPA), which confers invasive cells a substantial survival advantage during migration into the blood stream (1). Pharmacological blockade of P2RY12 with ticlopidine or apyrase was shown to reduce TCIPA *in vitro* (22, 23).

We have previously demonstrated that treatment with the P2RY12 antagonist, clopidogrel, decreased the size of pancreatic tumors and restored the hemostatic balance by preventing the accumulation of procoagulant cancer microparticles. It also reduced metastasis in mouse models of pancreatic cancer (24). These results were confirmed in studies done by other groups (25). However, a more profound understanding of the molecular mechanisms involved in the growth, spread, and pro-thrombotic effect of P2RY12 inhibition according to the cancer type is needed.

Using syngeneic ectopic and orthotopic pancreatic cancer mouse models, we compared the effect of aspirin and clopidogrel adjuvant treatment. While aspirin has proven its effects in cancer prevention, it does not seem to stop cancer progression once the disease is established in our pancreatic cancer mouse models. Clopidogrel has better anti-proliferative effects in our animal models, and both drugs significantly inhibited the metastatic spread of pancreatic cancer. Additionally, aspirin-treated mice had longer tail bleeding times than both the control and clopidogrel-treated groups; evidencing a higher platelet hemostatic inhibition. In a melanoma mouse model, clopidogrel-treated mice had an overall higher survival rate than the controls. We also demonstrated that pancreatic tumors, but not colorectal cancers, expressed clopidogrel target P2RY12. The activation of the P2RY12 on pancreatic cancer stimulates cell migration and proliferation. In conclusion, our study indicates a superiority of clopidogrel over aspirin as adjuvant therapy in pancreatic tumor progression and cancer-associated-thrombosis in mice. We therefore make the case that the role of P2RY12-inhibitors in cancer progression and cancer-associated-thrombosis warrants further investigation as a possible and new therapeutic target in pancreatic adenocarcinomas.

## Materials and Methods

### Antibodies and Reagents

Multiple antibodies were used in the different phases of this study. A rat anti-mouse GPIb-α antibody (0.2 µg/g body weight; Emfret analytics), a rat anti–mouse Ly-6G antibody (clone 1A8; 0.1 µg/g body weight; Becton Dickinson), and a labeled mouse anti-fibrin antibody were used as previously described (24, 26). To identify the cellular populations, we used an antibody directed against Human CD326/EPCAM labelled with PE DAZZLE (REF: B249041), antibody directed against Human CD41 labelled with PC7 (REF: 6607115), antibody directed against CD18 coupled to PE (REF: IM1570U), antibody directed against CD235 coupled with APC-Alexa Fluor 750 (REF: A89314), antibody directed against cytokeratin 7 (REF: 42900582), antibody directed against CD15 coupled to FITC (REF: E025953), antibody directed against CD326/EPCAM coupled to PE (REF: B222943), antibody directed against LGR5 coupled to biotin (REF: 130100852), antibody directed against PE coupled to microbeads (REF: 130100852), and antibody directed against biotin coupled to magnetic beads (REF: 130092322). We also used other antibodies to study both cellular and supernatant MP expression, such as an Anti-human CD326/EPCAM labeled with PE DAZZLE (REF: B249041), antibody anti human CD41 labeled with PC7 (REF: 6607115), antibody anti human CD235 labeled with APC-Alexa fluor 750 (REF: A89314), antibody anti human CD18 labeled with PE (REF: IM1570U), as well as antibody directed against P2RY12 coupled to PE from Biolegend (REF: 392103). We also used irrelevant antibodies labeled with the same fluorochrome as the antibodies. The microparticles were identified using AnnexinV-FITC in flow cytometry (REF: Fl18010). MP-count beads and Megamix-plus were from Biocytex and used to perform flow cytometry on Microparticles.

### Mice

Five-week-old, male, wild-type C57BL/6J mice and P2Y12KO mice (EMMA: 02303, B6;129P2Prep^tm1Dgen^/H) were obtained respectively from Janvier Lab and MRC Harwell. All animal care and experimental procedures were performed as recommended by the European Community guidelines and approved by the local ethical comity number 14 (protocol number 04280.03; 41-11102012 and APAFIS15334-2018060115491816). Only male mice were selected so that intravital microscopy could be used on the cremasteric veins and arteries (Intravital microscopy and laser-induced injury section).

### Human Primary Colorectal Cancer (CRC) Cell Isolation

Healthy and cancerous tissue biopsies were treated with the Miltenyi tissue dissociation kit (REF 130-095-929) according to the instructions of the manufacturer. Miltenyi Gentle MACS dissociator was used, and cell suspension was filtered before using magnetic separation columns (REF 130-042-401) and marking the cells with an antibody directed against CD326/EPCAM coupled to PE (REF: B222943) as well as antibody directed against LGR5 coupled to biotin (REF: 130100852). In sequence, we used anti-PE (REF 130-048-801) and anti-biotin microbeads (REF 130-090-485) to select intestinal stem cells in healthy and cancer tissues.

### Cell Culture

The mouse pancreatic cancer cell line Panc02 was generously given to Dr Laurence Panicot-Dubois by Dr Ruben Hernandez-Alcoceba (University of Navarra, Pamplona, Spain) (27). The mouse melanoma cancer cell line B16F10 (ATCC-CRL-6475) and the pancreatic cancer cell lines (mouse and human) Panc02, BXPC3, MiaPaCa2, SOJ6, and Panc1 and human colorectal cancer cell line HT29 were grown in RPMI-1640 and DMEM medium (Life Technologies) supplemented with 10% Fetal calf serum (PAA), 100 U/ml penicillin (Life Technologies), 100 µg/ml streptomycin (Life Technologies), and 0.1% fungizone (Life Technologies). The cells were grown at 37°C in a humidified atmosphere with 5% CO_2_.

### Cell Transfection

Panc02 and B16F10 cells were stably transfected with the pGL4.51[luc2/CMV/Neo] as previously described and respectively named Panc02-Luci (24) and B16F10-Luci. Panc02-GFP cells were obtained after transfection as previously described (28).

### mRNA Extraction

Human macroscopically healthy and cancerous colorectal tissue samples were obtained from the Department of Digestive Surgery service at the Timone Hospital, Marseille. The healthy and tumor tissue samples were transported in RNA later (REF: AM 7021) and were cut into small pieces with a scalpel and Trizol was added at a 1,000 µL per 100 mg ratio and were processed with the GentleMac RNA-01 program twice. The sample was placed on ice before adding chloroform (0.2 ml per 1 ml of Trizol used). The solution was centrifuged and the water phase containing the mRNA was recovered. Isopropanol was added at a 0.5 ml per 1 ml of Trizol used; the tubes were placed at -80°C for at least 12 hours. The mRNA was centrifuged to eliminate the supernatant liquid. mRNA was suspended in 70% ethanol and re-centrifuged. To verify the purity of the extracted mRNA, we used a Nanoview quantification machine to obtain the A260/A280 and A260/A230 ratios. The mRNA was conserved at -80°C. RNA Extraction from cultured cells were performed from cells with an 80% confluence. The cells were washed three times with ice-cold Mg^2+^ and Ca^2+^ free PBS (PBS ^-/-^) and the mRNA were isolated as described before.

### Reverse Transcription, Real Time PCR (RT-qPCR)

RT-PCR was executed using our tissue and cell isolated mRNA samples to obtain the complementary DNA chains. We prepared an RNA MIX using Oligod (T) 20 primer 50 μM, dNTP min 10 mM, and 2 micrograms of mRNA. The RT-PCR program was run (52°C for 10 minutes and 80°C for another 10 minutes). The resulting cDNA was analyzed with a Nanoview quantification machine. qPCR was performed on cDNA samples to establish the presence of P2Y1, P2Y12, and P2Y13 in our tissue samples. We used GAPDH, 18s, and HPRT-1 as the positive controls. The qPCR was run using a Step One Plus qPCR machine in 96-well micro plates. For each sample, 100 ng of cDNA was added to the PCR Mix. The plate was centrifuged at 1,200 rpm for 1 minute at 4°C. Afterwards, the microplate was subjected to qPCR for 1 h and 15 min (50°C for 2 minutes, 95°C for 10 minutes, and then 40 cycles of 95°C for 15 seconds and 60°C for 1 minute). The results were analyzed using the Step One software.

### PCR and Sequencing

A total of 20 µL of PCR products and 20 µL of the corresponding primer were sent to GATC Biotech. The FASTA sequence was checked (.seq file) and then, completed using the chromatogram (.abi file). The final nucleotide sequence was then compared on the NCBI database at http://blast.ncbi.nlm.nih.gov.

### Prime Flow Assay

We used the Thermo Fischer Scientific View RNA Cell Plus Assay kit (ref 88-19000) to preform Prime Flow on BXPC3 and HT-29 cells that were cultured in sterile Corning, tissue culture treated, flat bottomed 24-well plates (REF: 3526) with coverslips until they were at 80% confluence. We then proceeded to fixate and permeabilize the cells, antibody staining (antibody against CD31 coupled to PE), and target probe hybridization. The probes used were: P2RY1 coupled to Alexa-fluor 488 (Affymetrix ref: VA4-3087549); P2RY1 NEGATIVE coupled to Alexa-fluor 488 (Affymetrix ref: VA4-6000697); P2RY12 coupled to Alexa-fluor 647 (Affymetrix ref: VA1-3001186); P2RY12 NEGATIVE coupled to Alexa-fluor 647 (Affymetrix ref: VA1-6000696); P2RY13 coupled to Alexa-fluor 647 (Affymetrix ref: VA1-3009217); and P2RY13 NEGATIVE coupled to Alexa-fluor (Affymetrix ref: VA1-6000698). We then proceeded to amplify the signal and added DAPI 1/1000 (Invitrogen ref: 19882) to label the nuclei. The coverslips were then mounted, and we used a widefield fluorescent microscope with the Slidebook 6 software. The same software was used to analyze and quantify the fluorescence.

### Scratch Wound Healing Assay

BXPC3 or Panc02 cells were plated at 1 × 10 (5) cells/ml in a 12-well plate and incubated at 37°C 5% CO2 until a confluent monolayer was formed (48 h). A 1,000-µl pipette tip was used to create a scratch in the center of the monolayer and well. Cells were then gently washed three times with PBS -/- to remove detached cells. Cells were replenished with a serum-deprived medium alone or supplemented with ADP 20 µM and/or ticagrelor 10 µM. Images were taken using an inverted microscope at 4-hour intervals up to 16 h. Images were analyzed using the FIJI ImageJ software.

### Cell Proliferation Test

The cell proliferation assay was performed according to the instructions of the manufacturer (cat no. 11647229001; Roche Applied Sciences) using BXPC3 and Panc02 cells after a 16 h interaction with a serum-deprived medium alone or supplemented with ADP 20 µM and/or ticagrelor 10 µM.

### Cell Migration Test

For the cell migration test, 5 × 10 (4) BXPC3 or Panc02 cells were placed in 500 μL of RPMI medium without FBS or growth factors on a Boyden chamber insert (Falcon ref 353097) in a 24-well plate. The lower chamber was filled with 750 μL of either non-supplemented RPMI medium as the control or with ADP 20 µM and/or ticagrelor 10 µM. After 16 hours of incubation at 37°C 5% CO2, cells in the upper membrane were removed and those attached to the membrane underside were dyed with HOECHST 33342. The number of transmigrating cells was assessed by taking 10 random, non-overlapping images of each well using a Leica DMi8 fluorescence microscope.

### ADP Test

BXPC3 and Panc02 cells were tested for ADP secretion using the Sigma-Aldrich MAK033 according to the instructions. Colorimetric results were measured using the Promega Glomax Explorer with absorbance at 560 nm.

### Holotomography Microscopy

Nano Live CXA microscope was used to analyze Panc02 and BXPC3 live response to a serum-deprived medium supplemented with ADP 20 µM and/or ticagrelor 10 µM. Cells were recorded during 16 hours with an interval of 15 minutes. Experiments were performed in triplicate. Results were analyzed with the EVE Analytics (Nanolive).

### Ectopic Tumor Induction

Panc02-GFP cells were harvested as previously described and resuspended in PBS^-^/^-^. Mice (WT C57BL/6J or P2Y12KO) were injected subcutaneously in the right flank with a tumoral cell suspension (10^6^ cells in 100 µl of PBS^-^/^-^). When the tumor became palpable, measurements in two dimensions were performed with a digital caliper, and the volume of each tumor was calculated according to the following formula: π/6*(a*b^2^), where **a** is the largest diameter and **b** the smallest diameter of the tumor.

### Orthotopic Tumors Induction

Panc02-Luci or B16F10-Luci cells were harvested as previously described and resuspended in the RPMI medium. Mice were anesthetized with an intraperitoneal injection of ketamine (125 mg/kg; Panpharma), xylazine (12.5 mg/kg; Bayer), and atropine (0.25 mg/kg; Lavoisier). The peritoneal cavity was opened, and a mixture containing a Panc02-Luci cell suspension (2.10^6^ cells in 50 µl of RPMI medium) and 50 µl of Matrigel (BD Bioscience) was injected into the head of the pancreas. Temgesic (0.05 mg/kg) was subcutaneously administered to mice before the surgery and 12 hours later. Mice were daily observed and treated with Buprenorphine (up to 2.5 mg/kg) as soon as distinctive pain signs were detected.

### Mouse Treatment

Mice were orally administrated with clopidogrel (8 mg/kg, dilution in NaCl 0,9%), aspirin (10 mg/kg, dilution in water), or excipient (NaCl 0,9% or water) with a final volume of 200 µl. The concentrations of the drugs used in our article were calculated to induce a 50% reduction in thrombus formation following a laser-induced injury in healthy mice (29).

### Intravital Microscopy and Laser-Induced Injury

Intravital video microscopy of the cremaster muscle microcirculation was performed as previously described using the Intelligent Imaging Innovation system (21). Briefly, mice were anesthetized with an intraperitoneal injection of ketamine, xylazine, and atropine. A tracheal cannula was inserted, and the mouse maintained at 37°C on a thermo-controlled rodent blanket. A second cannula was placed in the jugular vein to inject antibodies to detect the thrombi formation and to keep the mouse anesthetized with regular injection of thiopental (15 mg/kg). After the scrotum was incised, the testicle and surrounding cremaster muscle were exteriorized onto an intravital microscopy tray. The cremaster preparation was perfused with a thermo-controlled (37°C) bicarbonate-buffered saline throughout the experiment. Vessel wall injury was induced with a micropoint laser system (Photonics Instruments) focused through the objective of the microscope, parfocal with the focal plane, and aimed at the vessel wall, as previously described (30). Micro-vessel data were obtained using an Olympus AX microscope with a 60x NA water-immersion objective. Digital images were captured with a Cooke Sensicam CCD camera in a 640 x 480–pixel format. The analyses were performed using the SlideBook software as described previously by Dubois et al. (31).

### *In Vivo* Bioluminescence Imaging

Prior to the *in vivo* imaging, the orthotopic cancer mice were given with an intraperitoneal injection of ketamine, xylazine and atropine (to anesthetize animals), and D-luciferin at 1.5 mg/kg (Promega). The luciferase activity of the tumor and/or organs (spleen, lungs, bowel, kidneys, and liver) was measured for 10 minutes using an *in vivo* bioluminescence imaging system (Biospace, Paris, France).

### Tail Bleeding Time

Mouse-tail bleeding times were determined as previously described (30). The investigator was blinded to the genotype or treatments. Briefly, a 3 mm portion of the distal tail was removed from mouse; the tail was immersed in isotonic saline (at 37°C), and the time to complete cessation of blood flow was recorded. The bleeding time was monitored for a maximum of 6 minutes.

### Immunofluorescence

Paraffin-tissue sections of human colon adenocarcinoma, normal colon, human moderately differentiated human pancreatic adenocarcinoma, and normal pancreas were obtained from Clinisciences. Each section was dipped in Diasolv (X0013) and rehydrated before being stained either with antibody directed against P2RY12 coupled to PE (from Biolegend REF: 392103) and antibody directed against CD41 coupled to APC (from Biolegend REF: 303709) or a PE coupled IGG (from Biolegend REF 400213) and APC coupled IGG (from Biolegend REF: 400120) for 2 h and Hoechst 3342 for 10 min. Samples were dehydrated and replaced in Diasolv before mounting with a coverslip using ProLong Gold Antifade Mountant (ThermoFisher REF P10144). Images were obtained with a Leica DMi8 fluorescence microscope, using a 40X objective with 10 random fields per sample before analyzing the area positive for each antibody. P2RY12 tissue-specific area was determined by subtracting the positive CD41 area from the positive P2RY12 area.

### Statistics

The significance was determined using Shapiro-Wilk normality test, followed by Wilcoxon’s rank-sum test, T-tests, or Mann Whitney. Bar graphs are shown as MEAN + SEM.

## Results

### CLOPIDOGREL (But Not Aspirin Treatment) Reduces the Tumor Growth, Metastasis Development, and Increases Survival in Mouse Cancer Models

**Figure 1:**
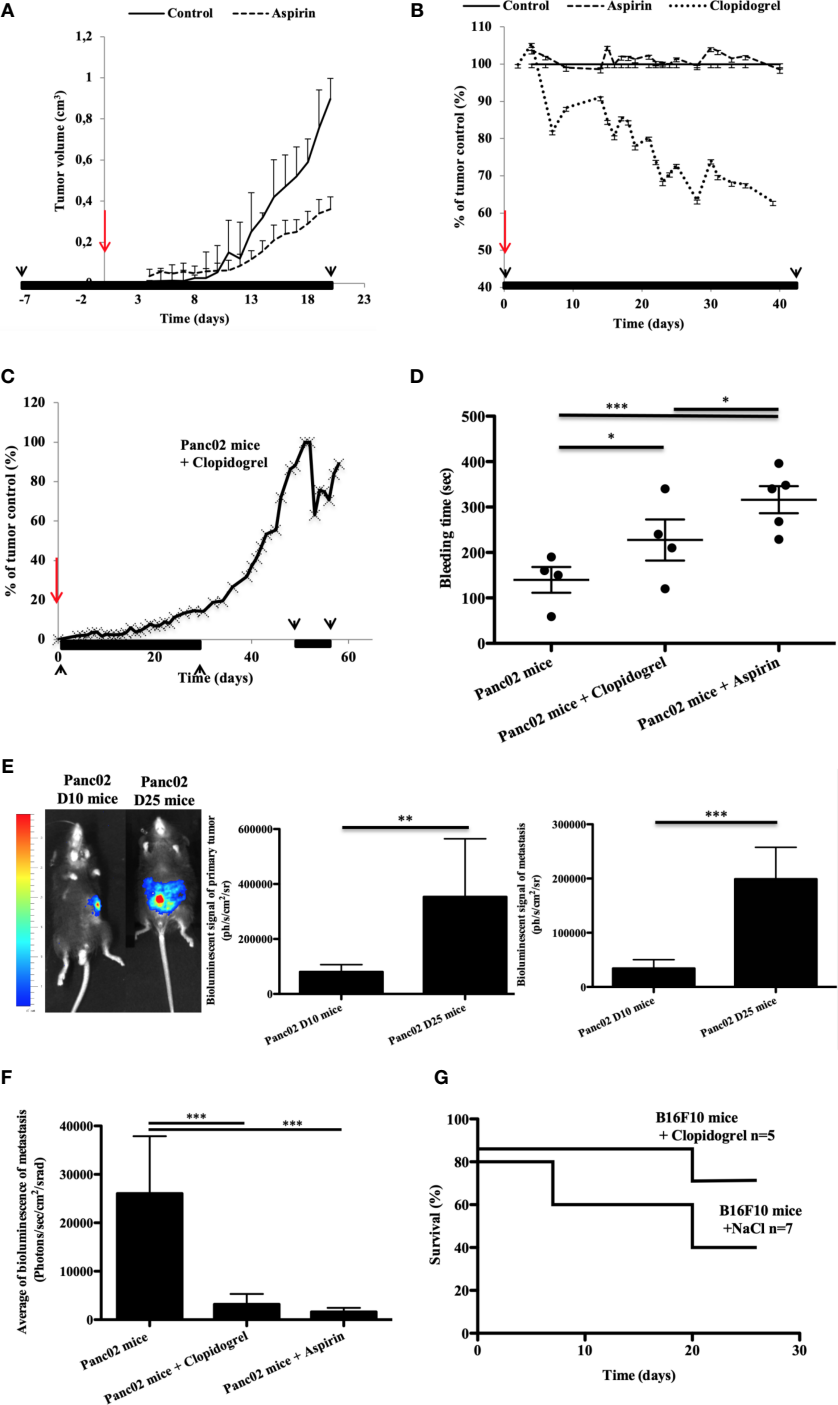
Clopidogrel, but not aspirin treatment, reduces the tumor growth in a syngeneic ectopic pancreatic cancer model in mice **(A–C)**. Clopidogrel strongly reduces the metastasis development and increases survival in orthotopic cancer models **(E–G)**. The tumor growth was evaluated in the tumor volume **(A)** or in % of the control tumor size **(B, C)** over time in ectopic pancreatic cancer mice treated or not with aspirin **(A, B)** or clopidogrel **(B, C)**. The period of the treatment is depicted in black lines and arrows whereas the time of cancer cells inoculation is in red arrows. **(D)** Graphic representation of Mean plus or minus of tail bleeding time for the cancer mice treated or not (n = 4) by clopidogrel (n = 4) or aspirin (n = 5). Statistical analysis was performed with a T-test. *P < 0.05; ***P < 0.0001. **(E)** Representative images of the bioluminescent signals at day 10 and 25 in orthotopic pancreatic cancer mouse model **(E)**, left panel. The mean of the bioluminescent signal was evaluated for the primary tumor and for the total metastasis (**E**, middle and right panels). **(F)** Quantification of metastasis development in mice treated by clopidogrel or aspirin in comparison to the control in a pancreatic orthotopic cancer model on isolated organs at day 30 after injection. **(G)** The survival curve was evaluated in a melanoma orthotopic mouse model, mice were treated or not (n = 7) with clopidogrel (n = 5). This experiment was performed with six mice per group. Statistical analysis was performed with a T-test. *P < 0,05, **P < 0,01, ***P < 0.0001.

We previously demonstrated that drugs targeting the platelet receptor P2RY12 reduced cancer progression and cancer-associated thrombosis in mice (24). Here, we aimed to determine the effects of aspirin and clopidogrel on the tumor growth in different pancreatic cancer mouse models (32). In accordance with what was reported in humans (33, 34), we found that aspirin used in a preventive setting (treatment stared 7 days before cancer inoculation) led to a 60% smaller tumor size at day 21 compared to the control (**Figure 1A**). However, when aspirin was administered as a curative treatment (treatment starting the day of inoculation), there was no significant decrease in the tumor size (**Figure 1B**). Different results were obtained using clopidogrel. When clopidogrel was administered as a curative treatment, there was an early inhibition of cancer growth with a 40% decrease in size compared to non-treated mice at the end point measurement (**Figure 1B**). Although, the tumor volume was strongly diminished by clopidogrel treatment (administered from day 0 to day 28 and from day 50 to day 57 post-inoculation), the cancer growth rate augmented immediately when the treatment was interrupted (**Figure 1C**).

We assessed the hemostatic action of aspirin and clopidogrel treatment by evaluating the tail bleeding time of the mice (35). Tail bleeding time assays demonstrated that aspirin treatment induced a strong and significant increase in the bleeding time compared to both the control and clopidogrel (**Figure 1D**). This indicates that platelet hemostatic function is likely more inhibited with aspirin treatment than with clopidogrel in our model.

To confirm the effect of anti-platelet P2RY12 drugs in the tumor growth, we next performed a syngeneic ectopic pancreatic cancer mouse model in P2RY12-deficient mice (EMMA: 02303, B6;129P2Prep^tm1Dgen^/H). In accordance with our previous results (24), the P2RY12-KO mice had significantly smaller tumors after 1 week and throughout the entire observation period than the WT controls (**Supplementary Figure 1**). Overall, the tumors in the control mice were almost double the size of the PRY12-KO. These observations confirm that P2RY12 has an important role in pancreatic adenocarcinoma development. Our results suggest that clopidogrel has a stronger potential to limit cancer development, and with a lower bleeding risk than aspirin.

Next, we aimed to compare the anti-metastatic potential of aspirin and clopidogrel in a cancer mouse model where metastasis formation occurs naturally (32, 36). We implanted Panc02-Luci (transfected mouse pancreatic cancer cell line) directly into the head of the pancreas of immunocompetent mice to create an orthotopic cancer mouse model. After inoculation, the bioluminescent signal of the primary tumor and the total spontaneous metastasis (Spleen, Lungs, Bowel, Kidneys, and Liver) were quantified on days 10 (D10) and 25 (D25) (**Figure 1E**). During cancer progression, we observed an increase in both primary tumor and metastasis bioluminescence signals (**Figure 1E**, middle and right panels). Both clopidogrel and aspirin treatment strongly decreased the development of spontaneous metastasis in comparison to the untreated mice (**Figure 1F**).

Interestingly, in an orthotopic murine model of melanoma, the action of clopidogrel on diminishing the tumor growth and metastasis led to an increase in the overall survival compared to the untreated mice (**Figure 1G**). These results show the need for more studies on the use of anti-P2RY12 drugs to treat cancer progression and metastasis development and to increase patient survival.

### Clopidogrel Inhibits Spontaneous Thrombosis During Advanced Stages of Pancreatic Cancer in Mice

**Figure 2:**
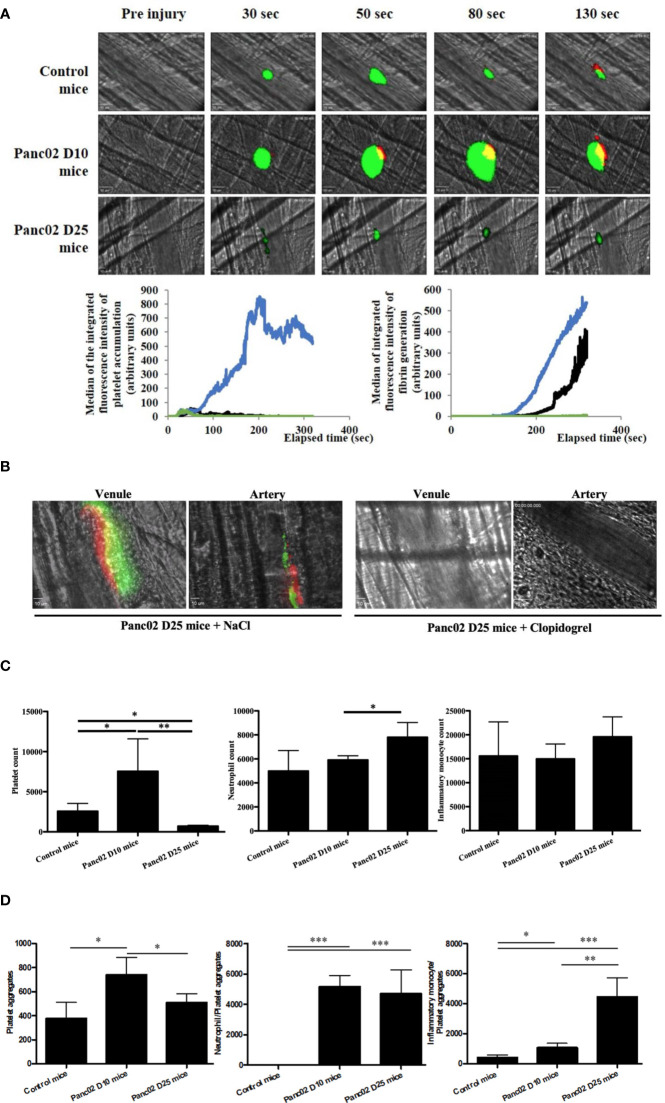
Clopidogrel inhibits the apparition of spontaneous thrombosis associated with advanced stage of pancreatic cancer in mice **(A, B)** and the cancer progression led to a variation of the platelets and immune cells counts **(C, D)**. **(A)** Representative images and quantification of platelet accumulation (in green) and fibrin generation (in red) at the site of thrombosis in Panc02 mice D10 (in blue, n=42 thrombi, 4 mice), Panc02 mice D25 (in green, n=46 thrombi, 4 mice) or in control mice (in black, n=48 thrombi, 5 mice). **(B)** Representative images of endogenous spontaneous thrombi with platelet accumulation (in green) and fibrin generation (in red) in venules and arteries of Panc02 cancer mice treated with NaCl (left panel) or clopidogrel (right panel). **(C)** Flow cytometry analysis of platelets (left panel), neutrophils (middle panel) and inflammatory monocytes (right panel) counts. *P < 0,05, **P < 0,01 **(D)** Flow cytometry analysis of platelet aggregates (left panel), neutrophils-Platelets aggregates (middle panel) and inflammatory monocytes-Platelets aggregates (right panel) aggregates *P < 0,05, **P < 0,01, ***P < 0,001.

Next, we studied the relationship between cancer progression and thrombus formation. We used an orthotopic model of pancreatic cancer to evaluate the kinetics of platelet accumulation and fibrin generation after a laser-induced injury in a vessel wall using real-time intravital microscopy (Furie Model) (31, 37). Remarkably, there was an evident pro-thrombotic phenotype at day 10 (Panc02 mice D10) after tumor implantation in comparison to the control mice (**Figure 2A**). Platelets and fibrin accumulated rapidly at the site of injury leading to larger thrombus sizes than in the control WT mice. In contrast, when we analyzed the thrombotic profile at day 25 after tumor implantation (Panc02 mice D25), they seem to have phenotypically changed to a pro-hemorrhagic state evidenced by the diminution of platelet and fibrin accumulation at the site of injury compared to both the Panc02 mice D10 and control (**Figure 2A**). These results are in accordance with the clinical data showing that advanced-stage cancer patients may have bleeding complications, which are mainly associated to disseminated intravascular coagulopathy (38–40).

We also observed a spontaneous formation of endogenous platelet and fibrin aggregates in advanced-stage-cancer mice (Panc02 mice D25) after cremaster preparation, without laser-induction, in both arteries and venules (**Figure 2B**). These spontaneous aggregates were inhibited after treatment with clopidogrel (**Figure 2B**, right panel). To our knowledge these spontaneous thrombi have not been described in literature yet, and reflect the clinical manifestations of cancer-associated thrombosis (38–40).

Consistent with our results of platelet and fibrin accumulation kinetics, at day 10 after pancreatic tumor implantation, there was an increase in the platelet count in comparison to the control mice (**Figure 2C**, left). Whereas, in advanced stage cancer, the platelet count was strongly decreased (**Figure 2C**, left). Regarding other cell types, neutrophil count was slightly increased in advanced stage cancer compared to both early stage and control, whereas no significant difference was observed for monocytes between the groups (**Figure 2C**, middle and right).

Of note, there were, in general, more spontaneous cellular aggregates formed in mice with pancreatic cancer than in the control counterparts, but the composition of the aggregates changed during disease progression (**Figure 2D**). At day 10 post-implantation, there were more platelet aggregates, but they diminished at day 25, the opposite being true for the platelet/inflammatory monocyte aggregates (**Figure 2D**).

We conclude that cancer progression could have induced a pro-inflammatory state, represented by the strong increase of inflammatory monocytes in the aggregates and circulating neutrophils in comparison to the controls (**Figure 2C**, middle and right; **Figure 2D**).

### P2RY12 Is Involved in Pancreatic Cancer Cell Migration and Proliferation, and its Expression is Specific to Pancreatic Adenocarcinoma

**Figure 3:**
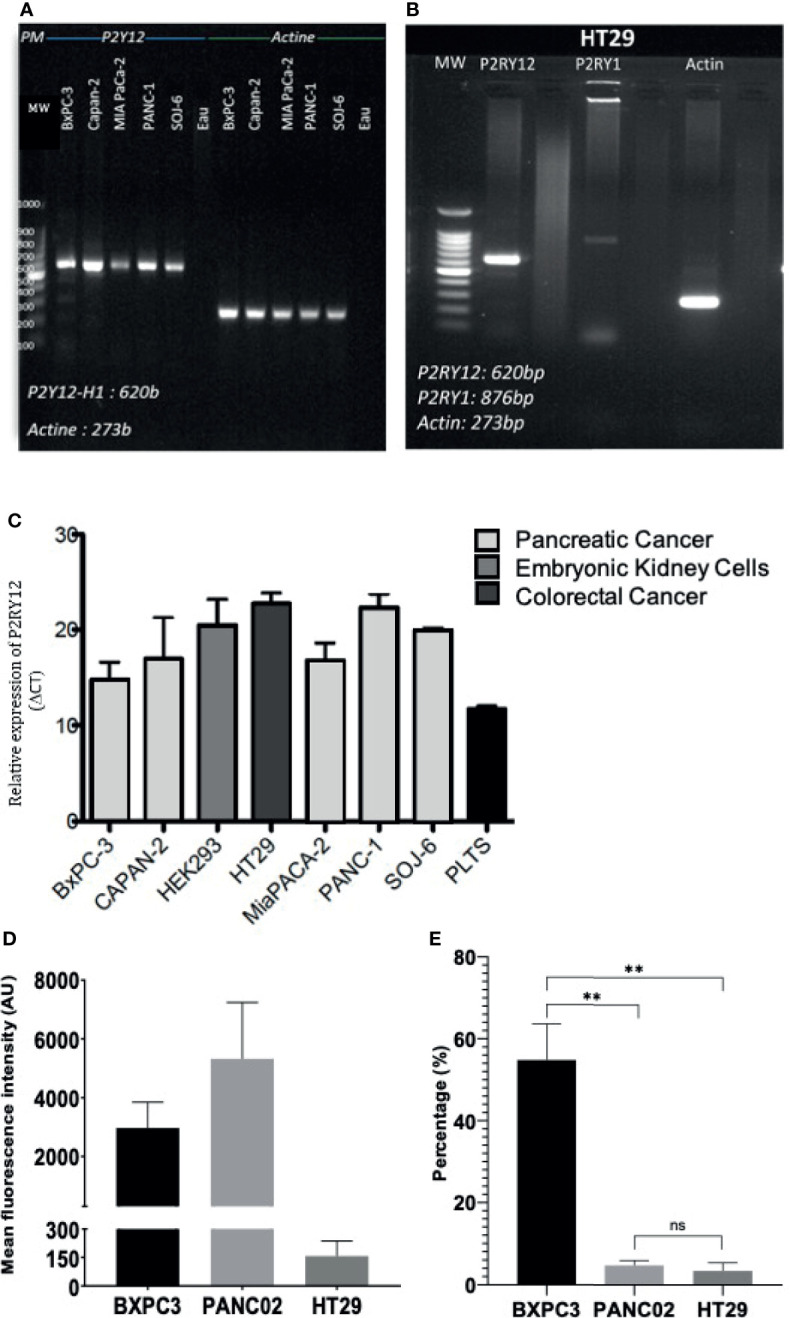
P2RY12 mRNA and protein expression in different cancer cell lines. **(A)** RT-PCR showing the P2RY12 mRNA in human and mouse pancreatic adenocarcinoma cell lines. **(B)** RT-PCR showing the P2RY1 and P2RY12 RNA in human colorectal adenocarcinoma cell line HT29. **(C)** Relative P2RY12 mRNA (delta CT) in different human and mouse cancer cell lines compared to platelets. **(D)** Mean Fluorescence of the P2RY12 protein in different pancreatic cancer cell lines BXPC3 and Panc02 as well as colorectal cancer cell line HT29. **(E)** P2RY12 protein expression normalized to the total cells in the samples analyzed with flow cytometry to show the relative population expression. Statistical test: Shapiro-Wilk and T-tests, **P < 0.001; ns, not- significant.

We next verified the presence of clopidogrel and ticagrelor target, P2RY12, on different tumor types. Using RT-PCR, we show that P2YRY12 mRNA was expressed in human pancreatic adenocarcinoma cell lines: BXPC3, CAPAN2, MiaPACA2, Panc1, and SOJ6 and colorectal cancer cell line HT29 (**Figures 3A, B**). All the cancer cell lines tested had a higher P2RY12 expression, ranging from 2X (Panc1) to 1.5X (BXPC3) than wild-type platelets (**Figure 3C**). These results were confirmed by flow cytometry analysis (**Figure 3D**). We correlated these flow cytometry results to the number of cells in each sample and found that the human pancreatic cancer cell line BXPC3 expressed P2RY12 more homogeneously as a population than the mouse pancreatic cancer cell line Panc02 (**Figure 3E**). To ensure that the P2RY12 was structurally functional, we sequenced the cDNA obtained from the isolated P2RY12-mRNA. We found that while all tested cell lines had point mutations, these were all silent and had no repercussion on the final synthesized protein (**Supplementary Figure 2**).

**Figure 4:**
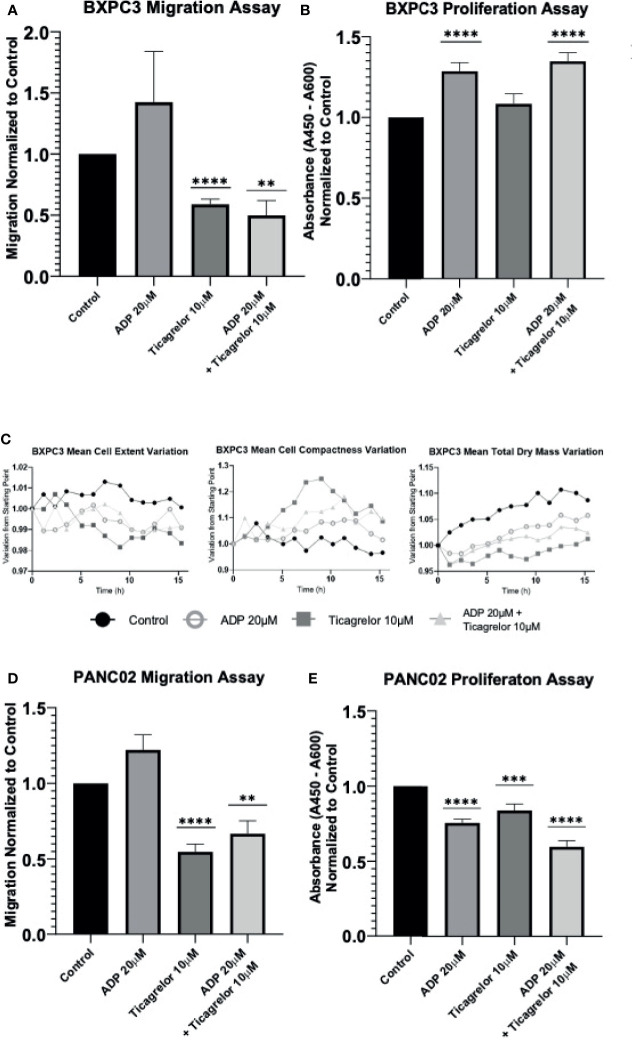
P2RY12 modulates pancreatic cancer cell migration and proliferation. **(A)** Migration assay using trans well chambers was performed with human pancreatic BxPC3 cells without treatment or stimulated with ADP (20 µM) or in presence of ticagrelor (10 µM) or treated with ADP in presence of ticagrelor at the same concentration as before. Graph represents the Mean +/- SEM for each condition normalized to control condition. **(B)** Proliferation assay of BxPC3 cells was tested using a commercially available BrdU kit. Cells were incubated without treatment or stimulated with ADP (20 µM) or in the presence of ticagrelor (10 µM) or treated with ADP in presence of ticagrelor. Graph represents the Mean +/- SEM for each condition normalized to control condition. **(C)** Using a holotomography microscope, we analyzed the changes in BxPC3 cell morphology such as Mean Cells Extent Variation, Mean Cell Compactness Variation, and Mean total dry mass Variation. These three parameters used to detect the apoptosis were analyzed in BxPC3 cell without treatment (Control; Full Black circle), BxPC3 cells stimulated with ADP at 20 µM (Open gray circle) or treated with Ticagrelor (10 µM) (Full square), and BxPC3 treated with ADP (20 µM) in the presence of ticagrelor (10 µM) (full triangle). The graphs represent the Mean of three experiments for each condition. **(D)** Migration assay of mouse pancreatic Panc02 cells. Cells were analyzed without treatment (control) or stimulated with ADP (20 µM) or in presence of ticagrelor (10 µM) or treated with ADP in presence of ticagrelor at the same concentration as before. Graph represents the Mean +/- SEM for each condition normalized to the control condition. **(E)** Panc02 cells were incubated without stimulation (control) or stimulated with ADP (20 µM) or in the presence of ticagrelor (10 µM) or treated with ADP in the presence of ticagrelor. Graph represents the Mean +/- EM for each condition normalized to the control condition. Statistical analysis was performed with Shapiro-Wilk and T-tests ****P < 0.0001, ***P < 0.001; **P < 0.01. All the experiments were performed in triplicate.

Next, we performed cell migration and proliferation assays to determine the effect of cancer cell P2RY12. In human PDAC cell line BXPC3, cell migration tests using trans-well chambers demonstrated a tendency to augment cell migration when P2RY12 was stimulated with ADP (**Figure 4A**). Interestingly, when P2RY12 was inhibited using ticagrelor, the migration effect significantly decreased to nearly half of the control (**Figure 4A**). Proliferation tests show a significant increase (1.5X) when in contact with ADP, even in the presence of P2RY12-inhibitor ticagrelor (**Figure 4B**).

We also studied the morphological changes in BXPC3 cells using a high-resolution holotomography microscope to determine whether ticagrelor treatment may induce apoptotic effects. Although some conformational changes related to apoptosis (cells become rounder, more compact, and their solid intracellular components decreased) were observed, these changes were not significant (**Figure 4C** and **Supplementary Video 1**).

In our mouse pancreatic cancer cell line Panc02, ADP stimulation of P2RY12 significantly enhanced cell migration, while its inhibition with ticagrelor significantly decreased migration by half compared to the control (**Figure 4D**). Interestingly, cellular proliferation was not enhanced in the presence of ADP, but it was significantly reduced when P2RY12 was blocked with ticagrelor (**Figure 4E**).

We determined that both human and mouse PDAC cell lines secreted 500-fold more ADP into their microenvironment than platelets, thus, it is possible that they are continuously activating their P2RY12 membrane receptors (**Supplementary Figure 3**) (21). We can conclude that P2RY12 plays an important role in the migration and proliferation of cancer cells.

**Figure 5:**
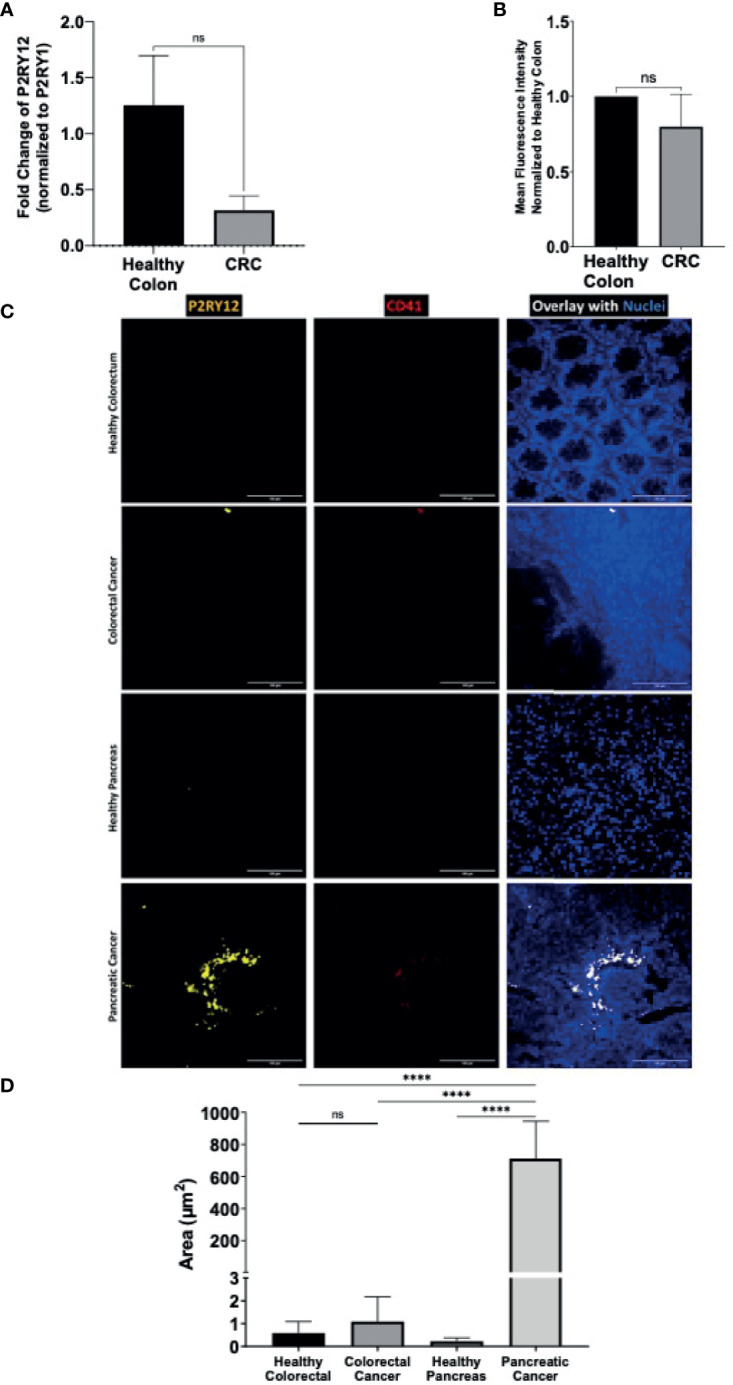
Cancer-tissue expression of P2RY12 is specific for pancreatic adenocarcinoma. **(A)** RT-qPCR of P2RY12 mRNA expression in human primary healthy colon and colorectal ADC. **(B)** Quantification of the P2RY12 protein expression by flow cytometry (mean fluorescence intensity) of LGR5+/EPCAM+ intestinal stem cell in human primary healthy colon and colorectal ADC. **(C)** Immuno-fluorescence of paraffin-embedded slides of primary human healthy (N = 3) and cancerous colorectal tissue (N = 3), primary human healthy (N = 3) and cancerous pancreatic tissue (N = 3) showing P2RY12 (yellow), CD41 (red), and Hoechst dyes (blue). Images obtained with a 40X objective, 10 images per sample. **(D)** Tissue-P2Y12 fluorescence area quantification. Statistical analysis was performed with Shapiro Wilk and Mann-Whitney tests ****P < 0.0001; ns, not- significant.

The next logical step was to determine if P2RY12 expression was specific to pancreatic adenocarcinoma, *versus* another digestive-tract cancer like colorectal adenocarcinoma. Colorectal cancer (CRC) is less known for developing thrombotic complications than pancreatic cancer (6, 9, 41). Although P2RY12 was detected at both the RNA (**Figure 5A**) and protein levels (**Figure 5B**) levels in tumor biopsies from patients with CRC, no significant difference was observed when compared to the healthy colorectal tissues (**Figures 5A, B**).

We used the immunofluorescence staining technique to determine and compare cancer and healthy tissue-specific P2RY12 presence. We stained human healthy and cancerous colorectal and pancreatic tissues with antibodies directed against P2RY12 and platelet marker CD41. Then, we determined endogenous P2RY12 expression by subtracting the area positive for platelet marker CD41.

Close to no P2RY12 was detected in the healthy colorectal samples (0.6+/-0.5 μm^2^; **Figures 5C, D** **)** or CRC samples (1.1+/-1 μm^2^ **Figures 5C, D**). There was no significant difference between these two kinds of tissue, suggesting a null colorectal P2RY12 expression between these two tissue types.

In the healthy pancreatic tissue, P2RY12 area was also very weak (0.23+/-0.15 μm^2^; **Figures 5C, D**). However, in the PDAC samples, the P2YR12 area was increased more than 3,000 times compared to the healthy pancreatic tissue (712+/-222 μm^2^) (**Figures 5C, D**).

Finally, comparing both cancer types, we detected a 650-fold increase of the P2RY12 positive area in pancreatic cancer compared to colorectal cancer (**Figure 5D**). Our results show that P2RY12 is not only expressed in platelets, but also in pancreatic cancer cells.

Taken together, our results show that clopidogrel or other P2RY12 inhibitors can significantly decrease the tumor growth and metastatic spread by inhibiting cell migration and proliferation. We also show that these antiplatelet drugs could be good candidates for treating Trousseau’s syndrome in cancer patients. Indeed, P2RY12 inhibitors not only stopped the formation of spontaneous aggregates in our advanced-stage-cancer model, but it did so with a smaller impact on the tail bleeding time than aspirin. We have also shown how P2RY12 expression could be specific to PDACs, opening new gates for researchers to determine its utility as a biomarker of malignant pancreatic disease or targeted cancer therapy.

## Discussion

There are many preclinical trials and meta-analysis demonstrating a biological rationale for the use of platelet antagonists as a preventive therapy for cancer. As our understanding of the intricate relationship between cancer cells and platelets gets deeper, so does the interest of using these drugs for cancer treatment. The use of aspirin to inhibit platelet aggregation has been largely studied as a preventive oncologic therapy (17, 42–44). Indeed, there are clinical meta-analysis suggesting that the use of a low-dose aspirin in the primary prevention reduces the risk of cancer diagnosis (45, 46). However, more studies are needed as more recent publications seem to show that not all cancers respond in a similar way to aspirin-mediated platelet inhibition; and that side effects of a long-term aspirin intake could be an important cause of patient morbidity (17, 18).

Another type of platelet-inhibitors, those that target the ADP-binding receptor P2RY12, have recently began taking the spotlight in cancer research. Preclinical trials have shown that P2RY12 inhibitors could reduce the tumor spread in melanoma, ovarian, breast, lung, and pancreatic cancers (24, 25, 47–51). A population-based historical cohort study from 2017 evidenced that a combined treatment with aspirin and clopidogrel was associated to a lower cancer risk in the population and proposed that clopidogrel alone could also reduce cancer incidence (52).

In this study, we first aimed to compare the potential anti-tumor properties of aspirin and clopidogrel (a commercially available P2RY12 inhibitor) in mouse syngeneic cancer models. In an ectopic pancreatic cancer mouse model, we show that aspirin treatment used in the primary prevention of cancer has a protective effect against tumor growth, but once the tumor is established, this effect is lost. On the other hand, clopidogrel used as a curative treatment of cancer appears to possess strong anti-tumor effects. In our orthotopic model we observed that both drugs significantly decreased tumor metastasis. Results were confirmed using P2RY12-KO mice, demonstrating that P2RY12 plays a role in PDAC development.

Tumor cells can activate platelets either in the microenvironment or in circulation through TCIPA; and so, it is logical to assume that antiplatelet drugs can also inhibit TCIPA and help diminish cancer spread (1, 2). Some cancer cells are known to be able to produce and secrete thromboxane A2 (TXA2) and ADP, which are potent stimulators of the TCIPA (53). In our study, we show how pancreatic ductal adenocarcinomas are capable of secreting 500-times more ADP than washed platelets, increasing local platelet activation and giving the tumor a proliferative advantage as well as increasing the local TCIPA.

Interestingly, the ADP scavenger apyrase and the ADP receptor antagonist 2-MeSAMP significantly affect the TCIPA induced by MCF7 human breast carcinoma cells *in vitro *(23). P2RY12 pharmacological blockade with ticlopidine and degradation of ADP by APT102 also reduced the TCIPA induced by neuroblastoma and Caco-2 intestinal cells *in vitro* (22, 54). The ADP scavenger apyrase reduced the TCIPA induced by MCF7 cells *in vitro*, while aspirin exerted no significant effect (53). These studies show that the P2RY12 blockade may have an important effect on diminishing the TCIPA by inhibiting platelet activation (1). We propose that this inhibition could also slow down cancer growth by inhibiting the P2RY12 expressed on the cancer cells themselves (47).

When activated, platelets can secrete transforming growth factor beta (TGF-β1) (55). TGF-β1 was demonstrated to synergistically activate the TGF-β/Smad and NF-κB pathway in cancer cells, resulting in their transition to an invasive mesenchymal-like phenotype, crucial for their invasiveness (56). Interestingly, it has been shown that P2RY12-deficient mice release less TGF-β1 from their activated platelets and had a reduced lung metastasis development compared to the controls (50). Clopidogrel-mediated blockade of P2RY12 may result in a decreased TGF-β1 secretion and diminished epithelial-mesenchymal transition (EMT).

Previous studies also demonstrated a role for platelets in promoting migration and adhesion to vessel wall *via* selectins. Indeed, we have already showed that treatment with clopidogrel prevents the P-selectin and integrin-dependent accumulation of cancer-cell derived microparticles and reduces tumor growth and metastasis *in vivo* (24). A similar effect is observed on cancer cells themselves, supporting our hypothesis of the anti-cancerous effects of P2RY12 inhibition on both platelets and cancer cells.

Platelet activation also induces angiogenesis in cancer through the release of pro-angiogenic and neo-vascularization factors (1). ADP-mediated vascular endothelial growth factor (VEGF) release was abrogated with cangrelor, another known P2RY12 antagonist, suggesting that P2RY12-inhibiton may reduce proangiogenic molecule release (57). Aspirin may also inhibit angiogenesis since TXA_2_ has been demonstrated to increase endothelial cell migration and angiogenesis in several *in vitro* and *in vivo* models (58). Additionally, it is known that activated platelets can mediate endothelial dysfunction through alterations in the nitric oxide-related pathways (59, 60). It has been demonstrated that the P2RY12 blockade synergizes with nitric oxide to further inhibit platelet activation and enhance arterial function (59, 60).

Trousseau’s syndrome is the leading cause of cancer-related death; and often, the first clinical sign of cancer (24, 61–63). It has been shown that in an advanced-stage cancer, the number of activated platelets not just increases (due in part to TCIPA), but platelets also become hyperreactive to agonists like ADP (64). To our knowledge, we have been the first to describe a mouse model of an advanced-stage cancer where the spontaneous formation of thrombotic aggregates was evidenced. Pancreatic cancer is particularly well-known for being pro-thrombotic (65, 66). Indeed, pancreatic cancer cells and their microvesicles can express tissue factor, podoplanin, and thrombin on their membrane (1). Here, we show that they also secrete large amounts of ADP into their microenvironment. This could be a tipping point for the hemostatic balance and may be the cause of the spontaneous thrombi we have observed. We believe this is a true clinical reflection of the Trousseau’s syndrome in pancreatic cancer. In this article, we demonstrate the potential benefits of treating this syndrome with known P2RY12 inhibitors, which have a lower risk for bleeding complications than aspirin treatment, as can be seen in our tail bleeding time results.

Interestingly, P2RY12 is also involved in the mediation of platelet inflammatory action through the release of inflammatory mediators (67). The inhibition of platelet-P2RY12 in a mouse model of abdominal sepsis was shown to reduce the release of pro-inflammatory mediators, notably IL-6, TNF-α, CCL4, and IL-1β (67, 68). Similar results were seen in a rat model of LPS-induced inflammation (69). A double-blinded randomized study comparing a placebo to the ticagrelor treatment in pneumonia showed that P2RY12 inhibition resulted in a significant decrease in plasma IL-6 levels (70). Since cancer is associated to a pro-inflammatory state, it is logical that P2RY12 inhibition could positively impact the tumoral microenvironment by reducing the pro-inflammatory signals (1, 71, 72).

Although several studies have analyzed the effect of P2RY12 inhibition on cancer progression, few studies have analyzed the presence or effect of this membrane receptor on cancer cells themselves. We found it very interesting that thienopyridine SR 25989 has anti-angiogenic properties and can inhibit lung metastasis of B16F10 mouse melanoma cells (73). This compound is an enantiomer of clopidogrel that inhibits P2RY12 but does not affect platelet aggregation (73). These results suggest that clopidogrel has an effect on cancer beyond the direct platelet inhibition.

Recently, it has been proposed that ticagrelor can be a new co-adjuvant therapeutic option in PDAC (47). Additionally, they established that P2RY12 activation on cancer cells induces cell proliferation through EGFR signaling (47). However, this study did not compare the level of P2RY12 expression according to the type of cancer nor platelets.

There are reports of the involvement of other members of the P2R receptor family in the progression of digestive tract cancers. For example, P2RY2, P2RY6, and P2RY11 are involved in the proliferation, metabolism, dissemination, apoptosis, and resistance to chemotherapeutic drugs in gastro-intestinal adenocarcinomas (74). To date, the differential expression of P2RY12 in different tumor-types and its role in cancer development has not been explored. We found that PDACs express 650-times more P2RY12 than colorectal ADC. Therefore, we propose that this molecule could be of interest as a novel marker of disease and treatment target.

Our results support that the P2RY12 blockade inhibits different molecular mechanisms that result in a diminished tumor growth and impaired metastatic spread. Clopidogrel can also diminish cancer-spread by impeding platelet activation and TCIPA. P2RY12 blockade also inhibits cancer-cell P2RY12 activation, resulting in a direct decrease in cancer-cell proliferation and migration. We found that this effect is specific for pancreatic cancer and is not relevant for colorectal cancer. Further studies will be required to determine which type of cancers express P2RY12 and could then be potentially treated with anti-P2RY12 drugs.

In conclusion, P2RY12 inhibitors diminish tumor growth and reduce metastasis formation and cancer-associated thrombosis. Moreover, it seems that the expression of P2RY12 in PDAC is tissue-specific and could represent a new potential therapeutic target. Still, more clinical studies are needed to fully understand the role of P2RY12 in different cancer types before including this molecule in future clinical trials.

## Data Availability Statement

The original contributions presented in the study are included in the article/**Supplementary Material**, further inquiries can be directed to the corresponding author.

## Ethics Statement

The studies involving human participants were reviewed and approved by Comité de Protection des Personnes Sud-Mediterranée II. The patients/participants provided their written informed consent to participate in this study. The animal study was reviewed and approved by Local ethical comity number 14, Aix Marseille University.

## Author Contributions

AP-A performed the experiments, analyzed the data, and wrote the original draft. SM and LC performed the experiments. DM designed the experiments. CD and LP-D designed the experiments analyzed the data and wrote the manuscript. All authors contributed to the article and approved the submitted version.

## Funding

This work was granted by French health governmental institute (INSERM) and by Aix-Marseille University. Ana-Luisa Palacios-Acedo was supported by the PAUSE program (College de France- PARIS).

## Conflict of Interest

The authors declare that the research was conducted in the absence of any commercial or financial relationships that could be construed as a potential conflict of interest.

## Publisher’s Note

All claims expressed in this article are solely those of the authors and do not necessarily represent those of their affiliated organizations, or those of the publisher, the editors and the reviewers. Any product that may be evaluated in this article, or claim that may be made by its manufacturer, is not guaranteed or endorsed by the publisher.

## Supplementary Material

Supplementary Figure 1 A syngeneic ectopic model with tumor growth to show that P2RY12-KO mice develop smaller tumors (WT N=5) (P2RY12-KO N=8). Statistical analysis: area under the curve and T test P= 0.0673.

Supplementary Figure 2 P2RY12 sequencing shows only silent mutations in tested cell lines.

Supplementary Figure 3 Mouse and human pancreatic cancer cell lines secrete significantly more ADP than human or mouse washed platelets, results are shown normalized to either human or mouse platelet ADP secretion.

Supplementary Video 1 Holo-tomographic video showing variation in total dry-mass in BPC3 cells either stimulated with ADP 20 μM or inhibited with Ticagrelor 10 μM. Changes shown as fold change normalized to starting point.
