# Supplementary figures and images for: P2RY12-Inhibitors Reduce Cancer-Associated Thrombosis and Tumor Growth in Pancreatic Cancers
Source: Front Oncol. 2021 Sep 13;11:704945. doi: 10.3389/fonc.2021.704945 (PMC8475274; doi:10.3389/fonc.2021.704945)

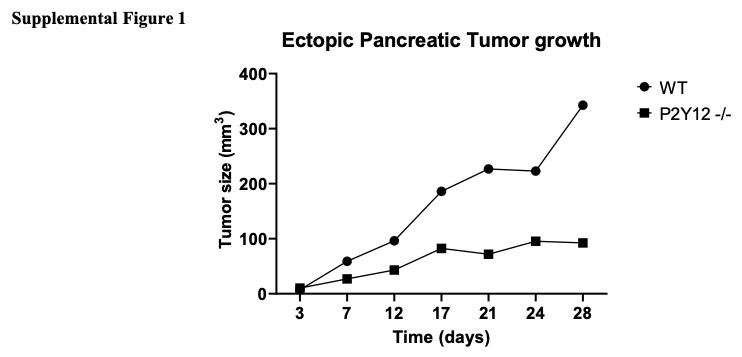

Supplement: Supplementary Figure 1 — A syngeneic ectopic model with tumor growth to show that P2RY12-KO mice develop smaller tumors (WT N=5) (P2RY12-KO N=8). Statistical analysis: area under the curve and T test P= 0.0673. [file Image_1.jpeg]

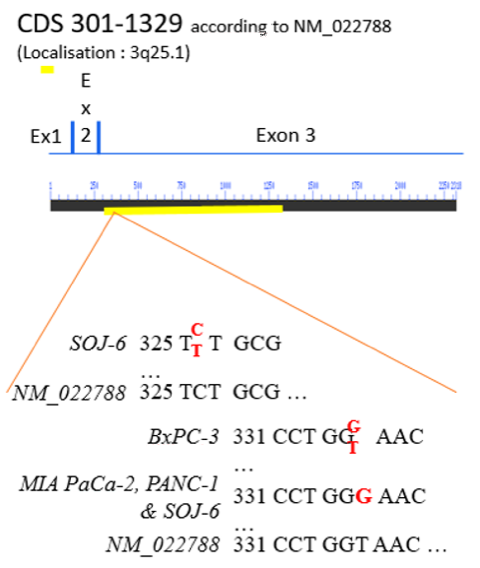

Supplement: Supplementary Figure 2 — P2RY12 sequencing shows only silent mutations in tested cell lines. [file Image_2.png]

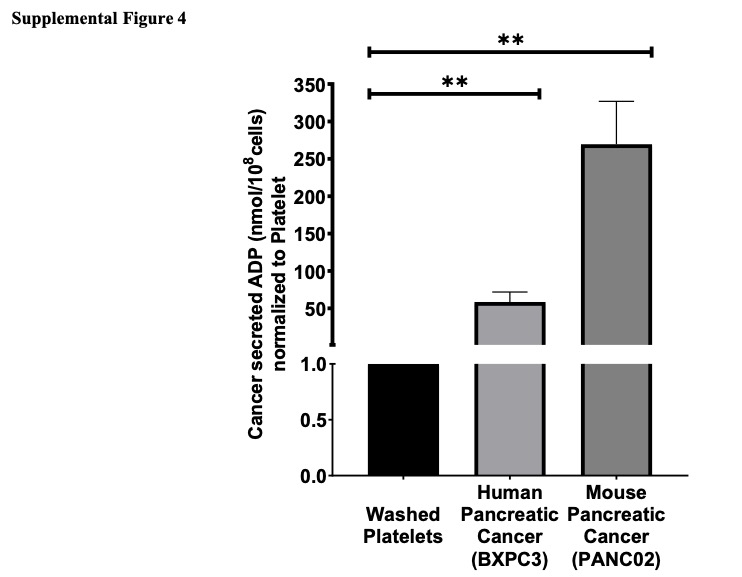

Supplement: Supplementary Figure 3 — Mouse and human pancreatic cancer cell lines secrete significantly more ADP than human or mouse washed platelets, results are shown normalized to either human or mouse platelet ADP secretion. [file Image_3.jpeg]
